# High-Quality Few-Layer Graphene on Single-Crystalline SiC thin Film Grown on Affordable Wafer for Device Applications

**DOI:** 10.3390/nano11020392

**Published:** 2021-02-04

**Authors:** Norifumi Endoh, Shoji Akiyama, Keiichiro Tashima, Kento Suwa, Takamasa Kamogawa, Roki Kohama, Kazutoshi Funakubo, Shigeru Konishi, Hiroshi Mogi, Minoru Kawahara, Makoto Kawai, Yoshihiro Kubota, Takuo Ohkochi, Masato Kotsugi, Koji Horiba, Hiroshi Kumigashira, Maki Suemitsu, Issei Watanabe, Hirokazu Fukidome

**Affiliations:** 1 Research Institute of Electrical Communication, Tohoku University, Sendai, Miyagi 980-8577, Japan; noriendo912@gmail.com (N.E.); tashey@riec.tohoku.ac.jp (K.T.); kensuwa@riec.tohoku.ac.jp (K.S.); b8tm2513@riec.tohoku.ac.jp (T.K.); r-kohama@riec.tohoku.ac.jp (R.K.); kazutoshi.funakubo@shi-g.com (K.F.); suemitsu@riec.tohoku.ac.jp (M.S.); 2 Shin-Etsu Chemical Co., Ltd., Chiyoda-ku, Tokyo 100-0004, Japan; s.akiyama@shinetsu.jp (S.A.); s_konishi@shinetsu.jp (S.K.); hiro_mogi@shinetsu.jp (H.M.); minoru.kawahara@shinetsu.jp (M.K.); mak_kawai@shinetsu.jp (M.K.); kubota-yoshihiro@shinetsu.jp (Y.K.); 3 Japan Synchrotron Radiation Research Institute, Sayo, Hyogo 679-5198, Japan; o-taku@spring8.or.jp (T.O.); kotsugi@spring8.or.jp (M.K.); 4 Photon Factory, Institute of Materials Structure Science, High Energy Accelerator Research Organization, Tsukuba, Ibaraki 305-0801, Japan; horiba@post.kek.jp (K.H.); kumigashira@tohoku.ac.jp (H.K.); 5 Institute of Multidisciplinary Research for Advanced Materials (IMRAM), Tohoku University, Sendai, Miyagi 980-8577, Japan; 6 National Institute of Information and Communication Technology, Koganei, Tokyo 184-8795, Japan; issei@nict.go.jp

**Keywords:** epitaxial graphene, SiC, affordable, transistor, terahertz

## Abstract

Graphene is promising for next-generation devices. However, one of the primary challenges in realizing these devices is the scalable growth of high-quality few-layer graphene (FLG) on device-type wafers; it is difficult to do so while balancing both quality and affordability. High-quality graphene is grown on expensive SiC bulk crystals, while graphene on SiC thin films grown on Si substrates (GOS) exhibits low quality but affordable cost. We propose a new method for the growth of high-quality FLG on a new template named “hybrid SiC”. The hybrid SiC is produced by bonding a SiC bulk crystal with an affordable device-type wafer and subsequently peeling off the SiC bulk crystal to obtain a single-crystalline SiC thin film on the wafer. The quality of FLG on this hybrid SiC is comparable to that of FLG on SiC bulk crystals and much higher than of GOS. FLG on the hybrid SiC exhibited high carrier mobilities, comparable to those on SiC bulk crystals, as anticipated from the linear band dispersions. Transistors using FLG on the hybrid SiC showed the potential to operate in terahertz frequencies. The proposed method is suited for growing high-quality FLG on desired substrates with the aim of realizing graphene-based high-speed devices.

## 1. Introduction

Graphene, which is a two-dimensional honeycomb lattice of carbon atoms, exhibits a linear band dispersion of π-electrons near the Fermi level [1,2,3,4,5]. This linear band dispersion results in excellent electronic properties, such as an extremely high carrier mobility [2,3,4], because of the vanishing of electron backscattering [6]. Furthermore, short-channel effects, which inhibit device integration, are suppressed in graphene, owing to its ultimate thinness [7]. These properties make graphene attractive for use in next-generation devices, such as field-effect transistors (FETs) [2,3,8,9] and lasers with frequencies in the terahertz range [10,11], which can provide the missing link between electronic information processing and optical communications [12].

For the realization of graphene-based devices, it is essential to develop methods for the scalable growth of high-quality graphene on semi-insulating device-type wafers [13]. One promising method involves growing high-quality few-layer graphene (FLG) on SiC bulk single crystals by annealing the crystals to sublimate the surface Si atoms [14,15]. SiC bulk crystals are used in power electronics and are now scaled up to 6 inches in diameter. In addition, this graphitization method eliminates the risk of metal contamination, which has an adverse effect on the functioning of devices such as p-n junctions. Although microscopic variations might exist in the thickness of the thus-grown FLG samples, the use of mesa on SiC substrates fabricated by conventional lithography techniques compatible with existing electronics (e.g., Si integrated circuits) has allowed for the realization of step-free SiC surfaces [16]. This has resulted in the growth of single-layer graphene without microscopic variations in thickness at the desired places for use in cases where high reliability is essential for device functioning, such as FET channels several microns in size [16]. Furthermore, in addition to single-layer graphene, homogeneous high-quality bilayer graphene can also be grown on SiC bulk crystals at temperatures higher than that for single-layer graphene growth [17]. Bilayer graphene on SiC bulk crystals exhibits a considerable bandgap under a vertical electric field [18]; this is predicted to ensure a high on/off ratio (>10^4^) in graphene-based tunnel FETs [19].

FLG grown on single-crystal SiC is hence promising owing to the above reasons and because of its compatibility with existing electronics (e.g., SiC-based power electronics). Unfortunately, the price of semi-insulating SiC bulk crystals is high. It has thus been difficult to balance affordability and quality when it comes to graphene. To achieve a balance between affordability and quality, we have developed a method for growing FLG on SiC thin films grown directly on Si by sublimating Si atoms on the SiC surface (GOS). The SiC thin films were grown directly on Si substrates using gas-source molecular beam epitaxy, with the source gas being monomethylsilane (H_3_C–SiH_3_) [20,21,22,23,24]. Moreover, combining this growth method and the three-dimensional micropatterning of the Si substrates opens a new way of controlling the formation of metallic and semiconductive FLG at desired locations on Si substrates on the nanoscale [25]. This method shows promise as a pathway towards the integration of electronic and photonic devices that employ graphene for the active layer.

Unfortunately, the quality of GOS remains low compared to that of FLG grown on SiC bulk crystals. This is because of the surface roughness of the 3C-SiC thin film, owing to the large mismatch in the lattice constants of SiC and Si [20,21,22,23,24].

Here we present a new method for the growth of high-quality FLG using a new template, which is termed “hybrid SiC” which consists of a high-quality SiC thin film by the peeling from a SiC bulk crystal, and an affordable device-type wafer. This method allows for the growth of high-quality FLG at affordable costs without any risk of contamination. FLG on the hybrid SiC showed excellent electronic properties. Field-effect transistors (FETs) using FLG on the hybrid SiC exhibited a simultaneous achievement of a large transconductance and drain current saturation. The FET demonstrates the potential to operate terahertz frequencies, and is promising for next-generation wireless communication.

## 2. Materials and Methods

**Figure 1:**
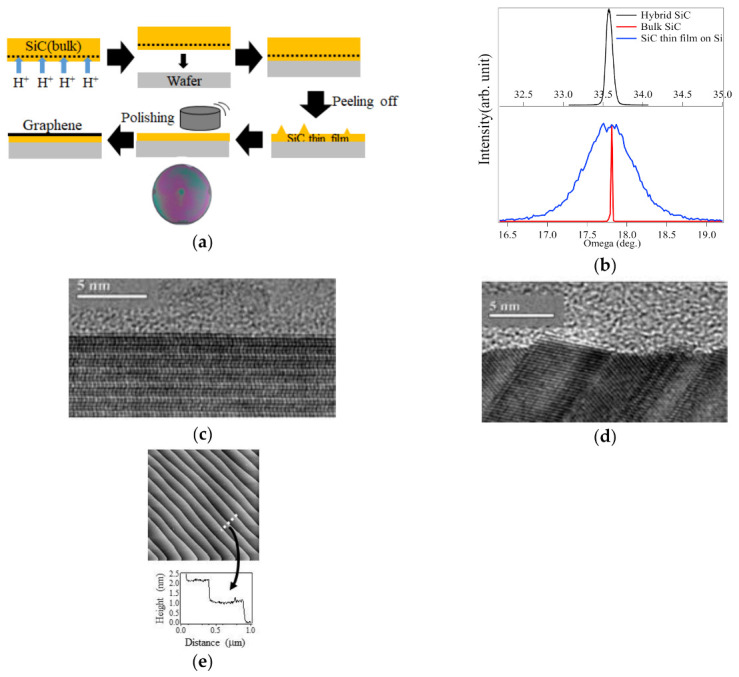
(**a**) Schematics of the fabrication of the hybrid SiC. (**b**) The x-ray diffraction of the hybrid SiC, a 3C-SiC thin film on a Si substrate. (**c**,**d**) are cross-sectional transmission electron micrographs of the hybrid SiC surface and the 3C-SiC thin film on the Si substrate, respectively. (**e**) The atomic force microscopy image of the hybrid surface. Inset shows the line profile across the dotted line.

The proposed method for growing high-quality FLG on the hybrid SiC is shown schematically in Figure 1a. The growth process is classified into two parts. The first part is the fabrication of the hybrid SiC [26]. First, H^+^ ions are implanted in the Si face of an on-axis 3-inch SiC bulk crystal to form a cut line (dashed line in Figure 1a), at which the SiC crystal can be separated easily. Then, the SiC bulk crystal is bonded to a device-type wafer (a 3-inch poly-SiC wafer was used in this study) with the intermediate layer, such as a SiO_2_ thin layer, which inhibits the channeling during the H^+^ implantation. During the bonding, the pressure was given by wafer’s own weight and the heating at ~600 K was performed. This is followed by the peeling of the SiC bulk crystal from the device-type wafer. The device-type wafer can be, for example, poly-SiC, Si, and sapphire. Finally, the surface is flattened by chemomechanical polishing (CMP). This SiC fabrication process is repeatable. Namely, multiple hybrid SiC can be produced by using a SiC bulk crystal. The thickness of the resulting SiC thin film on a device-type wafer was 300 nm. The second part is the graphitization of the hybrid SiC surface by annealing at 1850 K to sublimate the Si atoms on the surface in an argon atmosphere (1 bar), in a manner similar to epitaxial growth on SiC bulk crystals [14,16]. The graphitization was done by using radio frequency induction heating furnace installed in a super clean room (class < 10) of Research Institute of Electrical Communication (RIEC), Tohoku University.

X-ray diffraction (XRD) was used to characterize the crystallinity of the hybrid SiC, SiC bulk crystal, a 3C- thin film on a Si substrate. X-ray rocking curves were performed by using x-ray diffractometer (SuperLab, Rigaku, Tokyo, Japan) at RIEC. The curves were used to acquire XRD spectra of the hybrid SiC, and the 3C-SiC thin film on the Si substrate and the SiC bulk crystal. Atomic force microscopy was used to probe nanoscale surface roughness of the hybrid SiC. The AFM imaging was performed in air by using atomic force microscopy (Nanocute, Seiko Instruments, Chiba, Japan).

Raman spectroscopy was performed to evidence the high-quality of FLG on the hybrid SiC. Raman spectra were obtained by Raman microscopy (InVia^™^ confocal Raman microscope, Renishaw, Tokyo, Japan) at an excitation energy of 2.41 eV and lateral resolution of 1 μm.

Low-energy electron spectroscopy (LEEM) was performed to probe microscopic distribution of graphene layer number on the hybrid SiC. The LEEM imaging was done by using a low-energy electron microscope installed at BL 17 SU at SPring-8 (LEEM III, Elmitec, Clausthal-Zellerfeld, Germany) [27,28]. Before the LEEM imaging, the sample was degassed at 873 K under an ultrahigh vacuum (UHV) environment (<10^−7^ Pa). The layer number is digitally determined from the number of the dip in the electron reflectivity spectra which were obtained from the LEEM images [29]. The LEEM imaging was performed at room temperature under the UHV environment. By using the LEEM, microscopic LEED (μ-LEED) was carried out by collecting diffracted electrons from the region imaged by LEEM. The image was distorted a little because of the electron optics of the LEEM instrument [25].

Angle-resolved photoelectron spectroscopy (ARPES) using vacuum ultraviolet (VUV) radiation and core level spectroscopy using soft X-rays (SX) were performed at beamline BL-2 MUSASHI of Photon Factory, High Energy Accelerator Research Organization, Japan. Two undulators for the VUV and SX radiation arranged in a tandem alignment allowed for irradiation of the same position on the sample surface. The ARPES and C 1s core level photoelectron spectroscopy measurements were performed at photon energies of 80 and 800 eV, respectively, with the total resolutions being 20 and 200 meV, respectively. The sample was cooled to 20 K by using liquid He. The Fermi level of the sample was calibrated by measurements of a gold film that was electrically connected to the sample.

Hall-effect mobility measurement was performed to acquire carrier mobility and carrier density of FLG on the hybrid SiC and the SiC bulk crystal by using Van der Pauw method [30]. The carrier density (*n_s_*) is inversely proportional to the Hall voltage:

(1) $$n_{s}=\frac{BI}{eV_{H}}$$

where *V_H_*, *B*, *I*, *e* are the Hall voltage, magnetic field, current, and elemental charge, respectively. We calculated *n_s_* using Equation (1). The typical size of the pattern was 50 × 50 μm. The measurement was performed at room temperatures and under atmospheric environment at RIEC.

Transistors using FLG on the hybrid SiC was fabricated by using the cleanroom of RIEC. The gate insulator was formed by depositing on yttrium thin film, followed by oxidation in air at room temperatures. The gate length and width were 3.8 μm and 10 μm, respectively. The distance between the source and drain electrodes were 8 μm. The thickness of the yttrium oxide was 13.6 nm. Ti/Au thin film (5 nm/150 nm) was deposited on the sample for the formation of source, drain, and gate electrodes. The electrical characterization was performed by using semiconductor parameter analyzer (Agilent B1500A, Keysight).

## 3. Results

### 3.1. High Film Quality of the SiC Thin Film in the Hybrid SiC

The quality of SiC is a determining factor in the film quality of graphene layers grown on SiC. We examined the quality of the hybrid SiC by using XRD and X-TEM, and AFM. The crystallinity of the hybrid SiC was investigated by the x-ray rocking curves, as shown in Figure 1b. The full width at half maximum (FWHM) of the hybrid SiC (0.048) was order of magnitude smaller than that of 3C-SiC(111) (0.71), and comparable to that of the SiC bulk crystal (0.015). The obtained FWHM value clearly demonstrated that the crystallinity of the SiC thin film in the hybrid SiC was greatly improved, compared to that of the 3C-SiC thin film on Si(111), and comparable to that of SiC bulk crystal.

The atomistic surface morphology was examined by cross-sectional transmission electron microscopy (X-TEM) (Figure 1c), and compared with that of the 3C-SiC thin film grown directly on a Si substrate (Figure 1d). The X-TEM image of the 3C-SiC thin film (Figure 1d) shows that the grains were separated by grain boundaries, and the size of grains was on the order of 10 nm, as observed in the previous report [31]. On the other hand, the X-TEM image of the hybrid SiC (Figure 1c) confirms its single-crystalline nature; the grain boundaries are barely visible. Furthermore, the hybrid SiC had wide terraces with regularly spaced steps (Figure 1d). Thus, the hybrid SiC has a higher bulk and surface qualities, compared to that of the 3C-SiC thin film grown on the Si substrate, and comparable to that of the SiC bulk crystal.

### 3.2. High Film-Quality FLG Growth on the Hybrid SiC

The high quality of growth substrates promises high quality of materials on it. In fact, we demonstrated the high quality growth of FLG on the hybrid SiC by using C 1*s* core level photoelectron spectroscopy, X-TEM, and Raman spectroscopy, as shown below.

**Figure 2:**
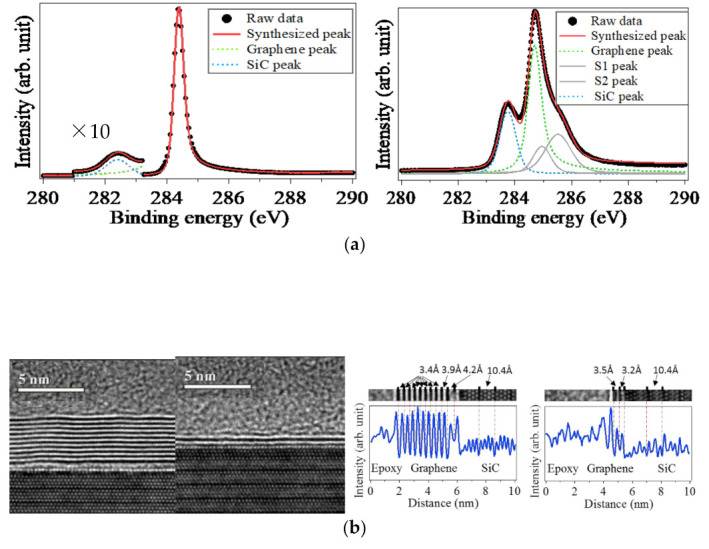

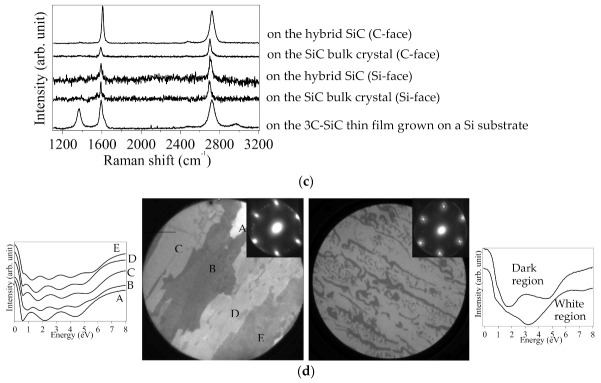
(**a**) C 1s core level spectra of graphene on the C-face (left) and the Si-face (right) hybrid SiC, respectively. (**b**) Transmission electron micrographs and line profiles of the interfaces between the C-face (left) and Si-face (right) hybrid SiC. (**c**) Raman spectra of graphene grown on the C-face of hybrid SiC and of the SiC bulk crystal, Si-face of hybrid SiC and the SiC bulk crystal, and the 3C-SiC thin film grown on the Si substrate from the top to the bottom, respectively. (**d**) LEEM images and electron spectra of graphene on the C-face (left) and Si-face (right) hybrid SiC, respectively. The field of view is 30 μm, and the energy of the incident electron for the LEEM imaging is 2 eV. The insets are μ-LEED taken from the observed regions.

The chemical compositions of FLGs on the Si-face and C-face hybrid SiC were revealed by the C 1s core level spectroscopy, as shown in Figure 2a. In the spectrum of FLG on the C-face hybrid SiC, the spectrum consists of two peaks due to FLG (∼284.5 eV) and SiC (∼282.3 eV) [22,23,25,32]. On the other hand, in the spectrum of FLG on the Si-face hybrid SiC, the spectrum contains the peaks labelled as peaks S1 and S2 due to the presence of the buffer layer [25,31], in addition to the peaks due to FLG and SiC. These results are consistent with the previous study on the graphene growth on the Si-face and C-face SiC bulk crystals [32,33]. This indicates that FLG on the hybrid SiC grows in a similar manner to that on the SiC bulk crystals.

Interfaces between FLGs and the Si-face and C-face hybrid SiC was investigated by X-TEM, as shown in Figure 2b. The X-TEM images clearly visualized the formation of the abrupt interfaces consisting of non-defective FLG and atomically flat Si-face and C-face hybrid SiC. The distance between the Si-face hybrid SiC surface and the nearest layer, the buffer layer on the Si-face SiC (3.2 Å) which was detected by the C 1*s* core level spectroscopy was shorter, compared to that between the buffer layer and the outmost layer, graphene layer (3.5 Å) [34,35]. The shortened distance arises from the covalent bonding between the SiC surface and the buffer layer, while there is no covalent bonding between graphene and the buffer layer, and between graphene layers [32,35]. On the other hand, the shortening of the distance between the nearest layer and the C-face hybrid SiC surface was not observed. This is explained by the absence of the buffer layer, which was elucidated by the C 1s core level spectroscopy (Figure 2a).

The film-quality of FLG was semiquantitatively investigated by using resonant Raman spectroscopy through real states of the valence and the conduction bands of FLG [36], as shown in Figure 2c. Only in the spectrum of graphene grown on the 3C-SiC thin film grown on the Si substrate, there appeared the D band that indicates the presence of defects in graphene. On the other hand, graphene grown on both the hybrid SiC and the SiC bulk crystal did not exhibit the D band. This result is indicative of the high quality of the FLG grown on the hybrid SiC. Semiquantitatively, the film-quality of the FLG was estimated in terms of the average grain size (*L_a_*) using the following empirical equation, which is based on the ratio of the intensities of the G (*I_G_*) and D bands (*I_D_*) [37]:

(2) $$L_{a}=\frac{560}{E_{i}^{4}}\left(\frac{I_{G}}{I_{D}}\right)$$

where *E_i_* is the energy of the incident photons (2.41 eV). Then, *L_a_* was assumed to be ~1 μm for the FLG grown on the hybrid SiC. This value is two orders of magnitude larger than that for the FLG grown on 3C-SiC thin films grown on Si substrates (20 nm) and comparable to that of the FLG grown on SiC [38]. The LEEM observation of FLG (Figure 2d) definitely agreed with the evaluation by using Raman spectroscopy. The layer number was digitally determined from the number of the dip in the electron reflectivity spectra which were obtained from the LEEM observation [16,29]. Then, it was found that the domain size of FLG on the hybrid SiC was several hundreds of nanometers to several micrometers. The observed grain size is comparable to that of FLG on SiC bulk crystals [14,16,29,39]. Thus, we successfully grew high film-quality FLG on the hybrid SiC.

The growth mechanism is discussed here. The different growth mechanisms of FLG on the Si-face and C-face hybrid SiC reflects the layer number and stacking manner of FLG. The layer number of FLG on the C-face SiC was larger than that on the Si-face hybrid SiC, as shown by the X-TEM images, the photoelectron spectra, and the LEEM images. The stacking manner was probed by acquiring μ-LEED, as shown in the insets of Figure 2d. The μ-LEED of FLG on the Si-face hybrid SiC exhibited a set of hexagonal spots, while that on the C-face SiC exhibited satellite spots in addition to the intense twinned hexagonal spots. The results indicated that FLG was Bernal-stacked on the Si-face hybrid SiC, while that on the C-face hybrid SiC contained rotational stacking faults [25,40]. This difference is explained by the presence/absence of the buffer layer between FLG and the hybrid SiC. The structure of the buffer layer is similar to that of graphene, but is partially bonded to the surface of the Si-face SiC. The buffer layer works as a precursor of graphene on the Si-face hybrid SiC, as in the similar manner of FLG on the Si-face SiC bulk crystal [14,32,40]. Hence, more energy is needed to grow FLG on the Si-face hybrid SiC owing to the buffer layer, compared to that on the C-face SiC. Therefore, the layer number of FLG on the Si-face hybrid SiC was decreased, compared to that on the C-face hybrid SiC. Simultaneously, the buffer layer works as a good template for Bernal stacking of FLG on the hybrid SiC because of the covalent bonding between buffer layer and the Si-face hybrid SiC. Thus, the rotational stacking faults are suppressed for FLG on the Si-face hybrid SiC. The buffer layer is the key factor in the FLG growth on the hybrid SiC.

### 3.3. Electronic Structure of FLG on the Hybrid SiC

**Figure 3:**
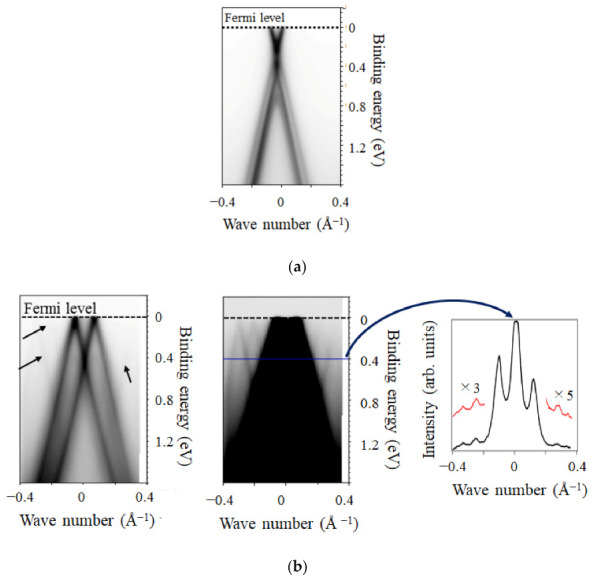
Angle-resolved photoelectron spectra images of few-layer graphene (FLG) on (**a**) the Si-face hybrid SiC and (**b**) the C-face hybrid SiC, respectively. In (**b**), the images with different contrasts and the line profile of the intensity at the binding energy of 0.4 eV for the sake of clarity of the faint bands.

The high-quality FLG grown on the hybrid SiC promises the linear band dispersion [41]. To confirm whether this was indeed the case, we recorded the ARPES spectra of the FLG sample on the Si-face and C-face hybrid SiC, as shown in Figure 3. On the Si-face hybrid SiC, the bands were energetically split (Figure 3a). As shown in LEEM image, monolayer and bilayer graphene domains exist on the Si-face hybrid SiC, and bilayer graphene is Bernal-stacked. Bernal stacking produces an interlayer interaction between graphene layers [42]. The interlayer interaction between graphene layers in bilayer graphene splits bands energetically, as observed in previous reports of the Bernal-stacked bilayer graphene on the Si-face SiC bulk crystal [18]. Thus, the band structure of graphene on the Si-face hybrid SiC reflects the presence of monolayer and Bernal-stacked bilayer graphene.

On the other hand, the band structure of graphene on the C-face SiC (Figure 3b) differs from that on the Si-face. In contrast to those on the hybrid Si-face, bands were not energetically split, but multiple bands appeared at different wave vectors. In addition to intense bands, faint bands existed, as indicated by the arrows in the ARPES image (left, in Figure 3b). Although these bands are faint, the presence of the faint bands is confirmed by looking at the image with the different contrast (middle, in Figure 3b) and the intensity line profile (right, in Figure 3b). The observations are explained by the fact that FLG was not Bernal stacked with rotational stacking faults on the C-face hybrid SiC [15,39], as shown in the μ-LEED (Figure 2d). The non-splitting of the bands is explained by the absence of Bernal stacking, which causes a negligible interlayer interaction.

### 3.4. Transport Properties of FLG on the Hybrid SiC

**Figure 4:**
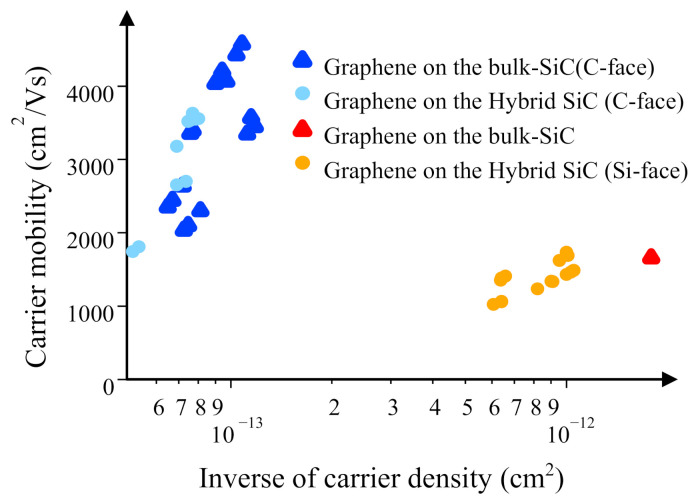
Hall-effect mobility measurements of FLG on the C-face and Si-face hybrid SiC and SiC bulk crystal at room temperatures.

The high film-quality and electronic structures promise excellent transport properties. This is corroborated by Hall effect measurements for the FLG films on the hybrid SiC and SiC bulk crystal at room temperatures, as shown in Figure 4. The mobilities of FLG on the SiC bulk crystal were also measured at room temperatures. It was found that the mobility of FLG on the hybrid SiC was comparable to that on a SiC bulk crystal. Furthermore, the carrier mobility increased, as the carrier density decreased. The similar trend is found in a previous report on the carrier mobility of FLG on the SiC bulk crystal [43]. Therefore, it is suggested that the electron transport can be limited mainly by surface polar phonons at room temperatures [43]. Furthermore, the carrier mobility of FLG on the Si-face hybrid SiC was found to be lower, compared to that on the C-face hybrid SiC. The similar trend was found for the carrier mobilities of FLG on the Si-face and C-face SiC bulk crystals [43]. The detailed mechanism for the lowering of the mobility of FLG on the Si-face hybrid SiC remains unclear. However, we guess that the buffer layer is responsible for the lowering of the mobility, as in the case of FLG on the Si-face SiC bulk crystal. One of the possible causes for this degradation is low-energy optical phonons related with the buffer layer [44]. In fact, we discovered the existence of an optical phonon mode associated with the interfacial Si atom possessing Si dangling bond [45], by using scanning inelastic tunneling microscopy, which probes vibrations at an atomic scale. This phonon induces the remote-interfacial phonon scattering and lowers the carrier mobility [46]. Thus, the high film quality of FLG on the hybrid SiC results in high carrier mobilities, comparable to those on the SiC bulk crystal.

### 3.5. Device Performances of FET Using FLG on the Hybrid SiC

**Figure 5:**
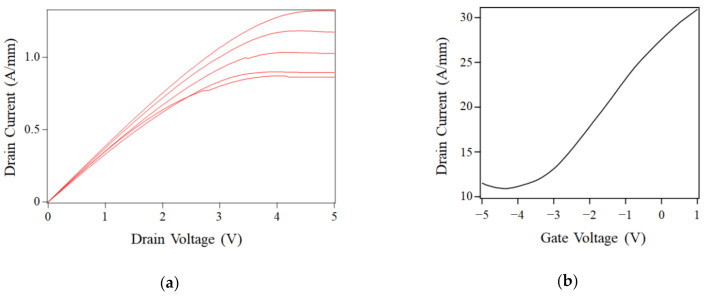
Electrical characteristics of a field-effect transistor using graphene grown on the hybrid SiC, (**a**) drain current–drain voltage with the gate voltages of 0 to 2 V (0.5 V step) and (**b**) drain current-gate voltage with the drain voltage of 10 mV.

The observed transport properties comparable to that of graphene on SiC bulk crystal promises excellent device performances. Actually, we succeeded in fabricating a high-performance FET using FLG on the hybrid SiC as a channel. This FET was electrically characterized, as shown in Figure 5. The FET exhibits the excellent DC characteristics of the drain current (*I_d_*)–drain voltage (*V_d_*) (Figure 5a), i.e., the simultaneous realization of a large transconductance (*g_m_*) of 307 mS/mm (maximum value) and quite a small drain conductance of 9.58 μS (minimum value), i.e., drain current saturation, as shown in Figure 5a. Besides, the FET showed an ambipolar behavior arising from the zero bandgap of graphene, as depicted in *I_d_*–gate voltage (*V_g_*) characteristics (Figure 5b). The carrier mobility of this FET was estimated to be 2.2 × 10^3^ cm^2^/Vs by using the direct transconductance method (DTM) [47]. The Dirac point where graphene was electrically neutral was around −4.2 V, meaning that graphene was n-doped.

The simultaneous realization of the large transconductance and the small drain conductance is significant in applications of FETs using graphene as channels (GFETs) [48]. A lot of previous studies reported that GFETs did not exhibit the drain current saturation because of the zero bandgap of graphene. Nanoribbonization was done to open the bandgap [49], but resulted in the degradations of the carrier mobility and the transconductance [49]. The drain current saturation of GFET without nanoribbonization was reported by few reports [50,51,52]. According to the previous reports [50,53], the drain current saturation of GFET can be obtained by the interplay of velocity saturation and density-of-states modulation. The interplay works well in case trap densities in the oxide and at the interface between graphene and oxide are mitigated [50]. This indicates the mitigating density of the trapped charges in our work. The high-*k* dielectrics including yttrium oxide can decrease the influence of the trapped charge [50]. Thus, we achieved the drain current saturation without nanoribbonization by adopting the high-k dielectric yttrium oxide ultrathin film as the gate dielectrics for our GFET, as well as the high quality of graphene on the hybrid SiC.

The simultaneous achievement of the large *g_m_* and the drain current saturation promises excellent high-frequency characteristics of our GFET. We estimated the upper limit of two typical figures of merit of high-frequency performances of our GFET, cutoff frequency for current gain (*f_T_*) and maximum oscillation frequency for power gain (*f_max_*) by using equations [48]:

(3) $$f_{T}=\frac{g_{m}}{2\pi }\frac{1}{\left(C_{gs}+C_{gd}\right)\left(1+g_{ds}\left(R_{S}+R_{D}\right)\right)+C_{gd}g_{m}\left(R_{S}+R_{D}\right)}$$

(4) $$f_{max}=\frac{g_{m}}{4\pi C_{gs}}\frac{1}{\sqrt{g_{ds}\left(R_{i}+R_{S}+R_{D}\right)+g_{m}R_{G}\frac{C_{gd}}{C_{gs}}}}$$

The value of relative dielectric constant of the yttrium oxide on graphene used in this work is 15 [54]. *R_S_* and *R_D_* are the sum of the contact and access-region resistances in the source and drain side. The sum of *R_S_* and *R_D_* is 81.7 Ω, which was obtained by analyzing the *I_d_* − *V_g_* characteristics [55]. *C_gs_* and *C_gd_* are capacitances between gate and source or drain, respectively. The values of *C_gs_* and *C_gd_* are 44.3 × 10^−15^ F and 11.8 × 10^−15^ F, respectively. The sum of *C_gs_* and *C_gd_* is the gate capacitance in total (C_G_). *R_G_* is the gate resistance, and is 0.65 Ω. *R_i_* is the resistance of the ohmic channel between the source and the gate. The values of *R_G_*, *R_i_*, *C_gs_*, and *C_gd_* in this estimation were obtained by analyzing the S-parameter measurement of the transistor using graphene grown on a SiC(0001) substrate with almost the same device structure, with the equivalent circuit where the function of GFET is expressed by the combination of resistors and capacitors [48]. In the equivalent circuit for FETs, the gate capacitance in total is decomposed into *C_gs_* and *C_gd_* because FET is in general the three-terminal device where the gate is connected to both the source and the drain through the channel. Then, we could estimate the values of *f_T_* and *f_max_* to be 23 GHz∙μm and 3.2 × 10^2^ GHz∙μm, respectively. These estimated values are comparable to or larger than those previously reported experimental values [56]. Especially, the estimated values of *f_max_* is promising because the degradation of *f_max_* has been the obstacle toward the high-frequency device application of GFET. Our estimation demonstrates that FETs using graphene grown on the hybrid SiC as the channel can be the high-frequency transistor that operate terahertz frequencies at a conventional gate length of 100 nm with proper device fabrication processes including, such as a minimization of the access resistances [57].

## 4. Discussion

We established the high-quality growth of FLG on a substrate termed hybrid SiC, which consists of a high-quality SiC thin film uniformly transferred from a SiC single crystal onto a device-type wafer with an affordable cost, compared to the graphene growth method using SiC bulk crystals. The fabrication process of the hybrid SiC is repeatable. It is difficult to precisely estimate the number of the repetition times because it depends on the process conditions. However, assuming that the decreased thickness of the SiC bulk crystal is 1 μm during one fabrication process of the hybrid SiC, the repetition times of the fabrication process of the hybrid SiC is roughly to be more than 100 times, considering that the thickness of as-received SiC bulk crystal is several hundreds of micrometers. Therefore, roughly speaking, the material cost of FLG on the hybrid SiC can be two orders of magnitude smaller than that of the conventional growth of FLG using the SiC bulk crystal.

FLG on the hybrid SiC exhibited high carrier mobility, comparable to that of graphene grown on the SiC bulk crystal. Owing to the simultaneous achievement of the complete drain current saturation and the large transconductance, the GFET demonstrated the potential to operate in terahertz frequencies. Our work is promising for the next-generation wireless communication systems that are the basic infrastructure of the forthcoming smart societies. Furthermore, our work may allow for the monolithic integration of graphene-based devices and existing electronics, such as GaN-based high electron mobility transistors (GaN-HEMTs) which use SiC as substrates, owing to the degree of freedom afforded regarding the choice of wafer.

## Data Availability

Not Applicable.

## References

[1] Wallace P.R. (1947). The band theory of graphite. Phys. Rev..

[2] Novoselov K.S., Geim A.K., Morozov S.V., Jiang D., Zhang Y., Dubonos S.V., Grigorieva I.V., Firsov A.A. (2004). Electric field effect in atomically thin carbon films. Science.

[3] Berger C., Song Z., Li T., Li X., Ogbazghi A.Y., Feng R., Dai Z., Marchenkov A.N., Conrad E.H., First P.N. (2004). Ultrathin epitaxial graphene: 2D electron gas properties and a route toward graphite-based nanoelectronics. J. Phys. Chem. B.

[4] Castro Neto A.H., Guinea F., Peres N.M.R., Novoselov K.S., Geim A.K. (2009). The electronic properties of graphene. Rev. Mod. Phys..

[5] Someya T., Fukidome H., Ishida Y., Yoshida R., Iimori T., Yukawa R., Akikuno K., Yamamoto S., Yamamoto S., Yamamoto T. (2014). Observing hot carrier distribution in an *n*-type epitaxial graphene on a SiC substrate. Appl. Phys. Lett..

[6] Ando T., Nakanishi T., Saito R. (1998). Berry’s Phase and Absence of Back Scattering in Carbon Nanotubes. J. Phys. Soc. Jpn..

[7] Avouris P. (2010). Graphene: Electronic and Photonic Properties and Devices. Nano Lett..

[8] Schwierz F. (2010). Graphene transistors. Nat. Nanotechnol..

[9] Novoselov K.S., Fal′ko V.I., Colombo L., Gellert P.R., Schwab M.G., Kim K. (2012). A roadmap for graphene. Nature.

[10] Bonaccorso F., Sun Z., Hasan T., Ferrari A.C. (2010). Graphene photonics and optoelectronics. Nat. Photon..

[11] Boubanga-Tombet S., Chan S., Watanabe T., Satou A., Ryzhii V., Otsuji T. (2012). Ultrafast carrier dynamics and terahertz emission in optically pumped graphene at room temperature. Phys. Rev. B.

[12] Tonouchi M. (2010). Cutting-edge terahertz technology. Nat. Photon..

[13] Sutter P., Sutter E. (2013). Microscopy of Graphene Growth, Processing, and Properties. Adv. Funct. Mater..

[14] Emtsev K.V., Bostwick A., Horn K., Jobst J., Kellogg G.L., Ley L., McChesney J.L., Ohta T., Reshanov S.A., Röhrl J. (2009). Towards wafer-size graphene layers by atmospheric pressure graphitization of silicon carbide. Nat. Mater..

[15] Sprinkle M., Siegel D., Hu Y., Hicks J., Tejeda A., Taleb-Ibrahimi A., Le Fèvre P., Bertran F., Vizzini S., Enriquez H. (2009). First direct observation of a nearly ideal graphene band structure. Phys. Rev. Lett..

[16] Fukidome H., Kawai Y., Fromm F., Kotsugi M., Handa H., Ide T., Ohkouchi T., Miyashita H., Enta Y., Kinoshita T. (2012). Precise control of epitaxy of graphene by microfabricating SiC substrate. Appl. Phys. Lett..

[17] Lauffer P., Emtsev K.V., Graupner R., Seyller T., Ley L., Reshanov S.A., Weber H.B. (2008). Atomic and electronic structure of few-layer graphene on SiC(0001) studied with scanning tunnelling microscopy and spectroscopy. Phys. Rev. B.

[18] Ohta T., Bostwick A., Seyller T., Horn K., Rotenberg E. (2006). Controlling the electronic structure of bilayer graphene. Science.

[19] Michetti P., Cheli M., Iannaccone G. (2012). Model of tunneling transistors based on graphene on SiC. Appl. Phys. Lett..

[20] Miyamoto Y., Handa H., Saito E., Konno A., Narita Y., Suemitsu M., Fukidome H., Ito T., Yasui K., Nakazawa H. (2009). Raman-scattering spectroscopy of epitaxial graphene formed on Si substrate. e-J. Surf. Sci. Nanotechnol..

[21] Fukidome H., Miyamoto Y., Handa H., Suemitsu M. (2010). Epitaxial growth processes of graphene on silicon substrates. Jpn. J. Appl. Phys..

[22] Fukidome H., Abe S., Takahashi R., Imaizumi K., Inomata S., Handa H., Saito E., Enta Y., Yoshigoe A., Teraoka Y. (2011). Controls over structural and electronic properties of epitaxial graphene on silicon using surface termination of 3C-SiC(111)/Si. Appl. Phys. Exp..

[23] Fukidome H., Takahashi R., Abe S., Imaizumi K., Handa H., Kang H.-C., Karasawa H., Suemitsu T., Otsuji T., Enta Y. (2011). Control of epitaxy of graphene by crystallographic orientation of a Si substrate toward device applications. J. Mater. Chem..

[24] Fukidome H., Kawai Y., Handa H., Hibino H., Miyashita H., Kotsugi M., Ohkochi T., Jung M.-H., Suemitsu T., Kinoshita T. (2013). Site-selective epitaxy of graphene on Si wafers. Proc. IEEE.

[25] Fukidome H., Ide T., Kawai T., Shinohara T., Nagamura N., Horiba K., Kotsugi M., Ohkochi T., Kinoshita T., Kumighashira H. (2014). Microscopically-Tuned Band Structure of Epitaxial Graphene through Interface and Stacking Variations Using Si Substrate Microfabrication. Sci. Rep..

[26] Kawai M., Kubota Y. (2014). The Method for Producing Nanocarbon Film and Nanocarbon Film.

[27] Fukidome H., Kotsugi M., Nagashio K., Sato R., Ohkouchi T., Itoh T., Toriumi A., Suemitsu M., Kinoshita T. (2014). Orbital-Specific Tunability of Man-Body Effects in Bilayer Graphene by Gate Bias and Metal Contact. Sci. Rep..

[28] Hasegawa M., Tashima K., Kotsugi M., Ohkochi T., Suemitsu M., Fukidome H. (2016). Inhomogeneous Longitudinal Distribution of Ni Atoms on Graphene Induced by Layer-Dependent Internal Diffusion. Appl. Phys. Lett..

[29] Hibino H., Kageshima H., Maeda F., Nagase M., Kobayashi Y., Yamaguchi H. (2008). Microscopic thickness determination of thin graphite films formed on SiC from quantized oscillation in reflectivity of low-energy electrons. Phys. Rev. B.

[30] Kim K.-S., Park G.-H., Fukidome H., Someya T., Iimori T., Fumio K., Matsuda I., Suemitsu M. (2018). A table-top formation of bilayer quasi-free-standing epitaxialgraphene on SiC(0001) by microwave annealing in air. Carbon.

[31] Handa H., Takahashi R., Abe S., Imaizumi K., Saito E., Jung M.-H., Ito S., Fukidome H., Suemitsu M. (2011). Transmission Electron Microscopy and Raman-Scattering Spectroscopy Observation on the Interface Structure of Graphene Formed on Si Substrates with Various Orientations. Jpn. J. Appl. Phys..

[32] Emstev K.V., Speck F., Seyller T., Ley L. (2008). Interaction, growth, and ordering of epitaxial graphene on SiC{0001} surfaces: A comparative photoelectron spectroscopy study. Phys. Rev. B.

[33] Riedl C., Coletti C., Starke U. (2010). Structural and electronic properties of epitaxial graphene on SiC(0001): A review of growth, characterization, transfer doping and hydrogen intercalation. J. Phys. D Appl. Phys..

[34] Norimatsu W., Kusunoki M. (2009). Transitional structures of the interface between graphene and 6H–SiC (0001). Chem. Phys. Lett..

[35] Norimatsu W., Kusunoki M. (2014). Epitaxial graphene on SiC{0001}: Advances and perspectives. Phys. Chem. Chem. Phys..

[36] Cançado L.G., Takai K., Enoki T., Takai K., Enoki T. (2006). General equation for the determination of the crystallite size L_a_ of nanographite by Raman spectroscopy. Appl. Phys. Lett..

[37] Tuinstra F., Koenig J.L. (1970). Raman spectrum of graphite. J. Chem. Phys..

[38] Caldwell J.D., Anderson T.J., Culbertson J.C., Jernigan G.G., Hobart K.D., Kub F.J., Tadjer M.J., Teesco J.L., Hite J.K., Mastro M.A. (2010). Technique for the Dry Transfer of Epitaxial Graphene onto Arbitrary Substrates. ACS Nano.

[39] Hibino H., Kageshima H., Maeda F., Nagase M., Kobayashi Y., Kobayashi Y., Yamaguchi H. (2008). Thickness Determination of Graphene Layers Formed on SiC Using Low-Energy Electron Microscopy. e-J. Surf. Sci. Nanotech..

[40] Hass J., de Heer W.A., Conrad E.H. (2008). The growth and morphology of epitaxial multilayer graphene. J. Phys. Condens. Matter..

[41] Rotenberg E., Bostwick A., Ohta T., McChesney J.L., Seyller T., Horn K. (2008). Origin of the energy gap in epitaxial graphene. Nat. Mater..

[42] McCann E., Fal’ko V.I. (2006). Landau-Level Degeneracy and Quantum Hall Effect in a Graphite Bilayer. Phys. Rev. Lett..

[43] Tedesco J.L., VanMil B.L., Myers-Ward R.L., McCrate J.M., Kitt S.A., Campbell P.M., Jernigan G.G., Culbertson J.C., Eddy C.R., Gaskill D.K. (2009). Hall effect mobility of epitaxial graphene grown on silicon carbide. Appl. Phys. Lett..

[44] Ray N., Shallcoress S., Hensel S., Pankratov O. (2012). Buffer layer limited conductivity in epitaxial graphene on the Si face of SiC. Phys. Rev. B.

[45] Minamitani E., Arafune R., Frederiksen T., Suzuki T., Shahed S.M.F., Kobayashi T., Endo N., Fukidome H., Watanabe S., Komeda T. (2017). Atomic-scale characterization, of the interfacial phonon in graphene/SiC. Phys. Rev. B.

[46] Fratini S., Guinea F. (2008). Substrate-limited electron dynamics in graphene. Phys. Rev. B.

[47] Brown M.A., Crosser M.S., Leyden M.R., Qi Y., Minot E.D. (2016). Measurement of high carrier mobility in graphene in an aqueous electrolyte environment. Appl. Phys. Lett..

[48] Schwierz F. (2013). Graphene Transistors: Status, Prospects, and Problems. Proc. IEEE.

[49] Wang X., Ouyang Y., Li X., Wang H., Guo J., Dai H. (2008). Room-Temperature All-Semiconducting Sub-10-nm Graphene Nanoribbon Field-Effect Transistors. Phys. Rev. Lett..

[50] Han S.J., Reddy D., Carpenter G.D., Franklin A.D., Jenkins K.A. (2012). Current Saturation in Submicrometer Graphene Transistors with Thin Gate Dielectric; Experiment, Simulation, and Theory. ACS Nano.

[51] Moon J.S., Curtis D., Bui S., Hu M., Gaskill D.K., Tedesco J.L., Asbeck P., Jernigan G.G., VanMil B.L., Myers-Ward R.L. (2010). Top-gated Epitaxial Graphene FETs on Si-Face SiC Wafers with a Peak Transconductance of 600 mS/mm. IEEE Electron Device Lett..

[52] Bai J.W., Liao L., Zhou H., Cheng R., Liu L., Huang Y., Duan X.F. (2011). Top-Gated Chemical Vapor Deposition Grown Graphene Transistors with Current Saturation. Nano Lett..

[53] Meric I., Dean C.R., Young A.F., Balitskaya N., Trembly N.J., Nuckolls C., Kim P., Shepard K.L. (2011). Channel Length Scaling in Graphene Field-Effect Transistors Studied with Pulsed Current-Voltage Measurements. Nano Lett..

[54] Wang Z., Xu H., Zhang Z., Wang S., Ding L., Zeng Q., Yang L., Pei T., Liang X., Gao M. (2010). Growth and Performance of Yttrium Oxide as an Ideal High-κ Gate Dielectric for Carbon-Based Electronics. Nano Lett..

[55] Kim S., Nah J., Jo I., Shahrjerdi D., Colombo L., Yao Z., Tutuc E., Banerjee S.K. (2009). Realization of a high mobility dual-gated graphene field-effect transistor with Al_2_O_3_ dielectric. Appl. Phys. Lett..

[56] Yu C., He Z., Song B., Liu Q.B., Han T.T., Dun S.B., Wang J.J., Zhou C.J., Guo J.C., Lv Y.J. (2017). Improvement of the Frequency Characteristics of Graphene Field-Effect Transistors on SiC substrate. IEEE. Electron Dev. Lett..

[57] Jung M.-H., Park G.-H., Yoshida T., Fukidome H., Suemitsu T., Otsuji T., Suemitsu M. (2013). High-Performance Graphene Field-Effect Transistors with Extremely Small Access Length Using Self-Aligned Source and Drain Technique. Proc. IEEE.

